# Five Sesquiterpenoids from a Marine-Derived Fungus *Aspergillus* sp. Isolated from a Gorgonian *Dichotella gemmacea*

**DOI:** 10.3390/md8040941

**Published:** 2010-03-29

**Authors:** Mei-Yan Wei, Chang-Yun Wang, Qing-Ai Liu, Chang-Lun Shao, Zhi-Gang She, Yong-Cheng Lin

**Affiliations:** 1 School of Pharmacy, Guangdong Medical College, Dongguan, 523808, China; E-Mail: mywei95@126.com (M.-Y.W.); 2 Key Laboratory of Marine Drugs, Ministry of Education, School of Medicine and Pharmacy, Ocean University of China, Qingdao, 266003, China; E-Mail: liuqingai1986@126.com (Q.-A.L.); 3 School of Chemistry and Chemical Engineering, Sun Yat-sen University, Guangzhou, 510275, China; E-Mails: cesshzhg@mail.sysu.edu.cn (Z.-G.S.); ceslyc@mail.sysu.edu.cn (Y.-C.L.)

**Keywords:** phenolic bisabolane-type sesquiterpenoid, Aspergillus sp., gorgonian Dichotella gemmacea

## Abstract

Three new phenolic bisabolane-type sesquiterpenoids: (+)-methyl sydowate (**1**), 7-deoxy-7,14-didehydrosydonic acid (**2**), and 7-deoxy-7,8-didehydrosydonic acid (**3**), together with two known fungal metabolites were isolated from the fermentation broth of a marine-derived fungus *Aspergillus* sp., which was isolated in turn from a gorgonian *Dichotella gemmacea* collected from the South China Sea. Their structures were elucidated by combined spectroscopic methods, and the structure of **1** was further confirmed by single-crystal X-ray data.

## 1. Introduction

Marine-derived fungi have been recognized as a potential source of structurally novel and biologically potent metabolites, and a growing number of marine fungi have been reported to produce novel bioactive secondary metabolites [1–3]. Especially, the genus *Aspergillus* has been known to be a major contributor to the secondary metabolites of marine fungal origin, for example, four sesquiterpenoids with a unique nitrobenzoyl ester from *Aspergillus versicolor* [4], two modified cytotoxic tripeptides from *Aspergillus* sp. [5], a novel pentacyclic oxindole alkaloid from *Aspergillus tamari* [6], four prenylated indole alkaloids from *Aspergillus* sp. [7] and two cyclopentapeptides from Aspergillus versicolor [8].

**Figure 1:**
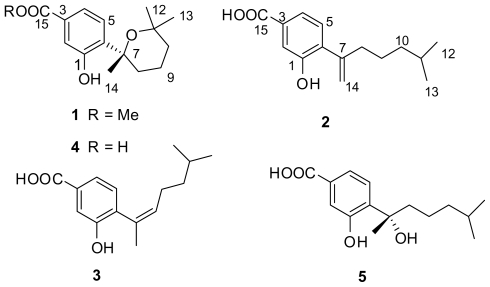
Structures of compounds **1**–**5**.

As part of our ongoing investigation into new bioactive natural products from marine fungi from the South China Sea, the gorgonian-derived fungus *Aspergillus* sp. attracted our attention because of the fact that a crude EtOAc extract of the fungal culture showed pronounced *in vitro* cytotoxicity against the A-549 human lung carcinoma cell line. Bioassay-guided fractionation of the extract led to the isolation of three new phenolic bisabolane-type sesquiterpenoids: (+)-methyl sydowate (**1**), 7-deoxy-7,14-didehydrosydonic acid (**2**), and 7-deoxy-7,8-didehydrosydonic acid (**3**), together with two known fungal metabolites: (+)-sydowic acid (**4**) [9,10], and (+)-sydonic acid (**5**) [11,12] (Figure 1). To date, this is the first report of natural products from a marine-derived fungus isolated from the fresh tissues of a gorgonian coral.

The EtOAc extract of a fermentation broth of the fungus *Aspergillus* sp. was subjected to silica gel column chromatography, Sephadex LH-20 and further semi-preparative HPLC, and this led to the isolation of compounds **1**–**5**. Their structures were elucidated by spectroscopic data, mainly 1D and 2D NMR spectra, and the structure of **1** was further confirmed by single-crystal X-ray data.

## 2. Results and Discussion

(+)-Methyl sydowate (**1**) was isolated as optically active colorless crystals ([α]^25^_D_ +24.7, CHCl_3_). A molecular formula of C_16_H_22_O_4_ was confirmed by HREIMS that displayed an [M]+ *m/z* of 278.1500 (calcd. 278.1513). The IR absorption bands indicated the existence of hydroxyl (3,230 cm^−1^) and ester (1,719 cm^−1^) groups. The ^1^H-NMR spectrum of **1** showed one exchangeable proton signal at *δ*_H_ 9.26 (s), one ABX spin system assignable to a 1,3,4-trisubstitued benzene ring at *δ*_H_ 7.10 (d, *J* = 7.8 Hz), 7.48 (d, *J* = 1.8 Hz) and 7.50 (dd, *J* = 7.8, 1.8 Hz) (Table 1). The ^13^C-NMR spectrum (Table 2) displayed 16 carbon signals, including those assigned to a carboxylic group (*δ*_C_ 166.9) and six aromatic carbons. With six degrees of unsaturation accounted for by the molecular formula, the structure of **1** was suggested to contain another ring, in association with a benzene ring and a carboxylic group. The NMR data of **1** were closely related to those of sydowic acid (**4**), a bisabolane-type sesquiterpenoid previously isolated from a terrestrial fungus *Aspergillus sydowi*.

**Figure 2:**
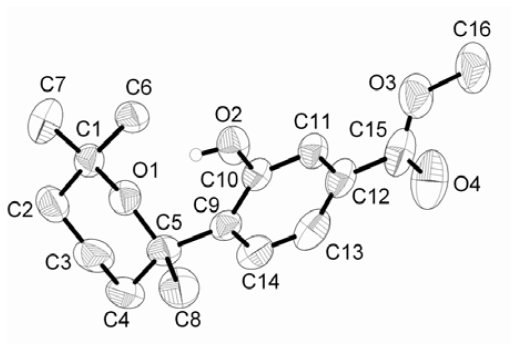
ORTEP drawing for methyl sydowate (**1**).

A comparison of NMR data showed that **1** was the methyl ester of **4**. The correlations from H_2_-8 through H_2_-9 to H_2_-10 in the COSY spectrum revealed the CH_2_-CH_2_-CH_2_ subunit in **1.** The C-12 and C-13 methyl singlets (*δ*_H_ 0.94 and 1.28) were determined to be germinal and attached to C-11 based on mutual HMBC correlations to each other and correlations from two methyl protons to C-10 and C-11. The connection between C-6 and C7 was established based on the HMBC correlations from H_3_-14 to C-6 and C-7, and H-5 to C-7. Crystals of **1** suitable for X-ray diffraction were obtained by slow evaporation of solution of **1** in methanol-DMF (1:1). The structure of **1** was further confirmed by a single-crystal X-ray analysis, and its ORTEP plot is depicted in Figure 2. Compound **1** exhibited a positive optical rotation similar to that of (+)-sydowic acid (**4**) [10], implying that its absolute configuration at C-7 was *R*.

7-Deoxy-7,14-didehydrosydonic acid (**2**) was isolated as a white powder, and its molecular formula of C_15_H_20_O_3_ was determined from HRESIMS data (found *m/z* 247.1331 [M – H]^−^, calcd. 247.1334). The molecular formula indicated **2** contained six degrees of unsaturation, which by interpretation of NMR data (Tables 1 and 2) could be attributed to four carbon-carbon double bonds, one carboxylic carbon, and one benzene ring. In the ^1^H-NMR spectrum, one ABX spin system assignable to a 1,3,4-trisubstitued benzene ring at *δ*_H_ 7.18 (d, *J* = 7.8 Hz), 7.66 (d, *J* = 1.2 Hz) and 7.65 (dd, *J* = 7.8, 1.2 Hz) was also observed. The ^1^H NMR spectrum revealed the presence of other signals including two doublet methyl groups [H_3_-12 (*δ*_H_ 0.83), and H_3_-13 (*δ*_H_ 0.83)], four methylenes [H-14a/14b (*δ*_H_ 5.45/5.19), H_2_-8 (*δ*_H_ 2.42), H_2_-9 (*δ*_H_ 1.39), and H_2_-10 (*δ*_H_ 1.18)], and one methine proton signal [H-11 (*δ*_H_ 1.51)]. From the ^13^C-NMR spectrum, one carboxylic carbon (*δ*_C_ 171.2), and eight sp^2^ carbons were observed. The NMR data of **2** closely resembled those of sydonic acid (**5**) previously isolated from a terrestrial strain of *A. sydowi* [11] and a marine fungus *Glonium* sp. [12]. The only significant differences in the ^1^H-NMR spectrum of **2** in comparison with **5** were two signals for H_2_-14 which were shifted downfield to *δ*_H_ 5.45 and 5.19 (instead of a methyl group at *δ*_H_ 1.68). The downfield shift observed for C-7 (*δ*_C_ 146.1 vs *δ*_C_ 79.3) and C-14 (*δ*_C_ 116.0 vs *δ*_C_ 21.7) in the ^13^C-NMR spectrum also reflected the presence of an exomethylene group rather than a methyl group connected to a quaternary carbonic carbon. Thus the gross structure of **2** was assigned as the 7,14-dehydration product of sydonic acid (**5**). The presence of the C-7/C-14 double bond was further supported by the HMBC correlations from H_2_-14 to C-6 and C-8, and from H_2_-8 to C-6, C-7 and C-14. These data allowed the complete structure of **2** to be assigned. Detailed assignments for carbons and protons were unambiguously accomplished by analysis of 2D NMR spectral data.

7-Deoxy-7,8-didehydrosydonic acid (**3**) was also obtained as a white powder with the same molecular formula, C_15_H_20_O_3_ (found *m*/*z* 247.1346 [M – H]^?−^, calcd 247.1334), as found for **2**. Detailed comparison of ^1^H- and ^13^C-NMR data of **3** (Tables 1 and 2) with those of **2** illustrated the presence of an olefinic bond at C-7/C-8 rather than C-7/C-14. This double bond was easily assigned since the signals for the methylene pair H_2_-8 and the terminal olefinic pair H_2_-14 were lost and replaced by single alkene signal at *δ*_H_ 5.76 (1H, t, *J* = 7.2 Hz, H-8) and one olefinic methyl group at *δ*_H_ 2.00 (3H, s, H-14). In a consistent fashion, the ^13^C-NMR spectrum showed an olefinic carbon for C-8 (*δ*_C_ 132.8) and an olefinic methyl group for C-14 at *δ*_C_ 24.7. Thus the structure of compound **3** was assigned as the 7,8-dehydration product of sydonic acid. In addition, the *Z*-geometry of the double bond was determined based on the chemical shift of the methyl carbon at the trisubstituted olefinic bond, which was observed at *δ*_C_ 24.7 (C-14) [13].

The origin of compounds **1**–**3** is a matter needing clarification. To determine if **1** was a natural product or it merely an artifact derived from methylation of (+)-sydowic acid during the isolation process, we analyzed the crude EtOAc extract by comparison of the retention times with that of pure sample of **1** by HPLC, using CH_3_CN/H_2_O (6:4) as a mobile phase. Compound **1** was clearly detected in the crude extract which had never been exposed to methanol, thus it seems very unlikely that **1** was obtained during the work-up. Since benzyl alcohols readily dehydrate under mild conditions, compounds **2** and **3** may be transformed from **5** during their isolation process. However, no dehydrated products were detected when **5** was dissolved in MeOH and left at room temperature for three days in the presence of hydrochloric acid (0.01 mol/L). Thus, dehydration of **5** during its isolation should have not occurred and correspondingly, compounds **2** and **3** should be considered true natural products.

The structures of compounds **4** and **5** were identified as (+)-sydowic acid [9,10], and (+)-sydonic acid [11,12], respectively, by comparison of their spectroscopic data with those in the literature. (−)-Sydowic acid was previously isolated from a terrestrial strain of *A. sydowi* [9], but its enantiomer (+)-sydowic acid was isolated for the first time as a natural compound in the present study. Sydonic acid was previously isolated in racemic form from the same species, *A. sydowi* [11], and interestingly, (+)-sydonic acid was also reported in 2009 from the fungus *Glonium* sp. collected from the Shirakami sea area [12].

A series of phenolic bisabolane-type sesquiterpenoids have been reported from different marine invertebrates, such as the marine sponges *Didiscus flavus* [14], *Parahigginsia* sp. [15] and *Epipolasis* sp. [16], and gorgonians *Pseudopterogorgia rigida* [17], *Muricia elongate* [18] and *Plexaurella nutans*[18], indicating that there only limited chemotaxonomic significance of these compounds in marine invertebrates. This type of compounds has recently also been reported from the bacterium CNH-741 and the fungus CNC-979, isolated from marine sediment [19]. In this study, five related sesquiterpenoids were also found from the marine-derived fungus *Aspergillus* sp. isolated from the gorgonian coral *Dichotella gemmacea* collected from the South China Sea. The findings of structurally related compounds from marine invertebrates and marine microorganisms could be used as circumstantial evidence to suggest that these compounds are acquired by the invertebrates from microbial symbionts or through their diet. It should be noted that the structures of compounds **2** and **3**, containing a double bond at C-7/C-14 or C-7/C-8, respectively, are unusual, since all of the previously known compounds are saturated at these positions. Recently, a strain fungus *A. sydowii*, isolated from healthy marine sponges *Spongia obscura* collected in Bahamian inshore waters, was reported as the causative agent of epidemics that affected gorgonian corals and had significantly affected their populations in the Caribbean Sea [20].

The bioactivity of compounds **1**, **4** and **5** were determined against *Staphylococcus aureus* and methicillin resistant *S. aureus* by the method as Fromtling *et al.* [21]. All of them exhibited weak antibacterial activity, with inhibition zones of 11, 7, 5 mm in diameter, respectively, at the concentration of 100 μg/mL. No inhibition, however, was observed for methicillin resistant *S. aureus* (kanamycin sulfate was used as the positive control with inhibition zones of 37 and 21 mm in diameter, respectively). Sydowic acid was reported as an antioxidant before [22]. No activities were evaluated for compounds **2** and **3** because of their low yields.

## 3. Experimental Section

### 3.1. General

^1^H- and ^13^C-NMR spectra were recorded on a JEOL Eclips-600 spectrometer. ESIMS and HRESIMS were measured on a Q-TOF Ultima Global GAA076 LC mass spectrometer. HREIMS were measured on a Thermo MAT95XP High Resolution mass spectrometer and EIMS on a Thermo DSQ EI-mass spectrometer. Optical rotations were measured in chloroform using a JASCO P-1020 digital polarimeter. IR spectra were measured on a Bruker VECTOR 22 spectrophotometer. Silica gel (Qing Dao Hai Yang Chemical Group Co.; 200–300 mesh), octadecylsilyl silica gel (Unicorn; 45–60 μm) and Sephadex LH-20 (Amersham Biosciences) were used for column chromatography (CC). Precoated silica gel plates (Yan Tai Zi Fu Chemical Group Co.; G60, F-254) were used for thin layer chromatography (TLC). Semi-preparative HPLC was performed on a Waters 1525 system using a semi-preparative C18 (Kromasil 7 μm, 10 × 25 mm) column coupled with a Waters 2996 photodiode array detector.

### 3.2. Fungal Material

The marine-derived fungus *Aspergillus* sp. was isolated from a piece of tissue from the inner part of the freshly collected gorgonian coral *D. gemmacea* (GX-WZ-20080034), which was obtained from the Weizhou coral reef in the South China Sea in September, 2008. The strain was deposited in the Key Laboratory of Marine Drugs, the Ministry of Education of China, School of Medicine and Pharmacy, Ocean University of China, Qingdao, PR China, with the access code ZJ-2008001. The fungal strain was cultivated in 30 L liquid medium (10.0 g of glucose, 2.0 g of yeast extract, 2.0 g of peptone in 1 L of seawater, in 1 L Erlenmeyer flasks each containing 400 mL of culture broth) at 27 °C without shaking for 30 days.

### 3.3. Extraction and Isolation

The fungal cultures were filtered through cheesecloth, and the filtrate (30.0 L) was extracted with EtOAc (2 × 30.0 L). The organic extracts were concentrated *in vacuo* to yield a yellow oily residue (2.50 g). This extract was chromatographed on a silica gel column using a stepwise gradient of petroleum ether–EtOAc to afford eight fractions (Fractions 1 8). Fraction 2 (0.35 g) was isolated by column chromatography on silica gel eluted with petroleum ether–EtOAc (8:2), and then subjected to Sephadex LH-20 chromatography eluting with mixtures of petroleum ether–CHCl_3_–MeOH (2:1:1) to obtain compound **1** (6.0 mg). Repeated chromatography of fraction 4 (0.22 g) using Sephadex LH-20 eluted with mixtures of CHCl_3_–MeOH (1:1) and petroleum ether–CHCl_3_–MeOH (2:1:1), then by semipreparative HPLC at a flow rate of 2.0 mL/min (6:4 MeOH/H_2_O) yielded compounds **2** (2.2 mg), **3** (1.5 mg), **4** (2.6 mg) and **5** (30.2 mg).

*(+)-Methyl sydowate* (**1**): colorless crystals; [α]^25^_D_ + 24.7 (*c* 0.030, CHCl_3_); UV (MeOH) *λ*_max_ 204.0, 238.0, 293.5 nm; IR (KBr) *ν_max_* 3230, 2972, 2932, 1719, 1573, 1281, 1201 cm^−1; 1^H-NMR and ^13^C-NMR see Tables 1 and 2; EI MS *m/z* 278 [M]^+^ (59), 263 (37), 260 (46), 245 (42), 231 (29), 217 (100), 203 (52), 195 (62), 192 (51), 189 (32), 179 (37), 173 (29), 161 (23), 145 (17), 131 (14), 69 (32); HREIMS *m/z* [M]^+^ 278.1500 (calcd for C_16_H_22_O_4_, 278.1513). Crystallizes in triclinic, space group *P*-1 with *a* = 7.0260(13) Å, *b* = 8.1016(15) Å, *c* = 13.939(3) Å, *α* = 87.266(2), *β* = 77.823(2), *γ* = 76.502(2)°, C_16_H_22_O_4_, *M_r_* = 278.34, *V* = 754.2(2) Å^3^, *Z* = 2, *D_c_* = 1.226 g/cm3, *F*(000) = 300, *μ* = 0.087 mm^−1^, the final *R* = 0.0461 and *wR* = 0.1095 for 5811 observed reflections (*I* > 2*σ* (*I*)). The crystallographic data for **1** have been deposited at the Cambridge Crystallographic Data Centre (CCDC No.738932).

*7-Deoxy-7,14-didehydrosydonic acid* (**2**): white powder; UV (MeOH) *λ*_max_ 209.8, 247.4, 300.6 nm; IR (KBr) *ν_max_* 3071, 2946, 2860, 1692, 1640, 1533, 1507, 1407, 1288, 1215, 764 cm^−1; 1^H-NMR and ^13^C-NMR see Tables 1 and 2; ESIMS *m/z* [M – H]^−^ 247; HRESIMS *m/z* [M – H]^−^ 247.1331 (calcd for C_15_H_20_O_3_, 247.1334).

*7-Deoxy-7,8-didehydrosydonic acid* (**3**): white powder; UV (MeOH) *λ*_max_ 206.3, 253.3, 304.2 nm; IR (KBr) *ν_max_* 3104, 3065, 3005, 1699, 1540, 1514, 1447, 1215, 758 cm^−1; 1^H-NMR and ^13^C-NMR see Tables 1 and 2; ESIMS *m/z* [M – H] ^−^ 247; HRESIMS *m/z* [M – H] ^−^ 247.1346 (calcd for C_15_H_20_O_3_, 247.1334).

### 3.4. Antibacterial activity

The compounds were tested against *S. aureus* and methicillin resistant *S. aureus* for their inhibitory activity. Antibacterial assays were performed using a modified version of the 2-fold serial dilutions method as Fromtling *et al.* [21].

## Figures and Tables

**Table 1:** ^1^H-NMR data (CDCl_3_) of **1**–**3** a.

position	1 *δ*_H_ (mult., *J* in Hz)	2 *δ*_H_ (mult., *J* in Hz)	3 *δ*_H_ (mult., *J* in Hz)
1	–	–	–
2	7.48, d (1.8)	7.66, d (1.2)	7.65, d (1.2)
3	–	–	–
4	7.50, dd (7.8, 1.8)	7.65, dd (7.8, 1.2)	7.67, dd (7.5, 1.2)
5	7.10, d (7.8)	7.18, d (7.8)	7.13, d (7.5)
6	–	–	–
7	–	–	–
8	2.43, ddd (13.8, 3.6, 0.6) 1.70, m	2.42, t (7.8)	5.76, t (7.2)
9	1.74, m 1.64, m	1.39, m	1.81, m
10	1.54, m	1.18, m	1.19, m
11	–	1.51, septet (6.6)	1.45, m
12	0.94, s	0.83, d (6.6)	0.77, d (6.6)
13	1.28, s	0.83, d (6.6)	0.77, d (6.6)
14	1.49, s	5.45, s 5.19, s	2.00, s
15	–	–	–
−OCH_3_	3.90, s	–	–
−OH	9.26, s	–	–

a Measured at 600 MHz.

**Table 2:** ^13^C-NMR data (CDCl_3_) of **1**–**3** a.

position	1	2	3
1	157.0, -C	152.4, -C	151.7, -C
2	118.3, CH	117.1, CH	116.6, CH
3	130.5, -C	129.4, -C	129.3, -C
4	120.7, CH	122.0, CH	122.3, CH
5	124.5, CH	128.2, CH	128.7, CH
6	136.0, -C	134.1, -C	133.7, -C
7	77.6, -C	146.1, -C	130.2, -C
8	33.8, CH_2_	37.7, CH_2_	132.8, CH
9	16.6, CH_2_	25.6, CH_2_	27.1, CH_2_
10	36.7, CH_2_	38.5, CH_2_	38.6, CH_2_
11	75.2, -C	27.8, CH	27.4, CH
12	24.7, CH_3_	22.5, CH_3_	22.3, CH_3_
13	31.9, CH_3_	22.5, CH_3_	22.3, CH_3_
14	31.3, CH_3_	116.0, CH_2_	24.7, CH_3_
15	166.9, -C	171.2, -C	170.6, -C
−OCH_3_	52.0, CH_3_		

a Measured at 150 MHz.
